# Prenatal Perfluorooctyl Sulfonate Exposure and Alu DNA Hypomethylation in Cord Blood

**DOI:** 10.3390/ijerph15061066

**Published:** 2018-05-24

**Authors:** Chen-Yu Liu, Pau-Chung Chen, Pei-Chen Lien, Yi-Peng Liao

**Affiliations:** 1 Institute of Environmental Health, College of Public Health, National Taiwan University, Taipei 10055, Taiwan; r01844006@ntu.edu.tw (P.-C.L.); katherinepope10@hotmail.com (Y.-P.L.); 2 Institute of Occupational Medicine and Industrial Hygiene, College of Public Health, National Taiwan University, Taipei 10055, Taiwan; pchen@ntu.edu.tw

**Keywords:** perfluoroalkyl substances, prenatal exposure, epigenetics, DNA methylation

## Abstract

Background: Perfluoroalkyl substances (PFASs) are stable and persistent in the environment, animals, and humans. PFASs can penetrate placenta and affect fetal growth. We investigated associations between prenatal exposure to perfluorooctanoic acid (PFOA), perfluorooctyl sulfonate (PFOS), perfluorononanoic acid (PFNA), and perfluoroundecanoic acid (PFUA) and global methylation levels. Specific Aims and Methods: The study used the subjects from Taiwan Birth Panel birth cohort study, including all pregnant women who gave birth between July 2004 and June 2005 in four hospitals in Taipei city and New Taipei City. A total of 363 mother-infant pairs were included in the final analyses. PFOA, PFOS, PFNA, and PFUA were measured by UPLC-MS/MS in cord blood. LINE-1 and Alu repeated elements from cord blood was used to represent global DNA methylation levels. Multivariable regression models were used to adjust potential confounders. Results: After controlling for potential confounders, each unit increase in the natural log-transformed PFOS exposure was associated with an adjusted OR of 1.72 (95% CI: 1.03, 2.88) for low Alu methylation level when dichotomized methylation level by medium. No significant effects between PFOA, PFNA, PFUA and methylation levels in the multivariable regression models were observed. Conclusions: Our findings suggest that prenatal PFOS exposure may be associated with low Alu methylation level.

## 1. Introduction

Perfluoroalkyl substances (PFASs), a class of man-made chemicals, are composed of completely fluorinated carbon chains with different functional head groups. PFASs are widely used in daily and industrial products because of their water-, oil-, stain and heat resistant properties, such as food wrappers and non-stick cookware. Due to the strong carbon-fluorine bond, PFASs are stable and persistent in the environment, including air dust, surface water and soil, and in many species, range from wildlife, plants and humans. Dietary intake has been suggested as the major exposure rout to PFASs in human. The mean elimination half-lives of serum PFASs in humans are 5.4 years for perfluorooctyl sulfonate (PFOS) and 3.8 years for perfluorooctanoic acid (PFOA) [1]. PFASs can penetrate placenta and make prenatal exposure of potential concern. Animal [2,3,4,5] and human studies [6,7,8] have reported PFOS and PFOA exposure, especially during pregnancy, were associated with adverse birth outcomes [4,6,9,10], allergic responses [11,12], and cancer through a non-genotoxic mechanism [13]. PFOS and PFOA are believed to act as non-genotoxic agents [14,15]. Many non-genotoxic xenobiotics have been found to induce toxicity by altering DNA methylation patterns [16,17,18]. The PFOS and PFOA related adverse effects may be induced through changing DNA methylation status. 

DNA methylation is a mechanism of epigenetic regulation and is essential for the development of mammals [19,20], involves the addition of a methyl group to the 5-position of a CpG dinucleotide [21]. Early embryonic development may be particularly susceptible to epigenetic dysregulation caused by environmental exposures because DNA methylation patterns are being established and cell division rates are high [22,23]. 

Change of DNA methylation level may affect genomic stability and gene expression without directly changing DNA sequence. Global DNA hypomethylation is generally associated with genomic instability and is commonly observed in many complex diseases [24,25]. Human genome is largely comprised of repetitive elements, including approximately 500,000 long interspersed nucleotide elements (LINE-1) and 1.4 million the short interspersed nucleotide elements Alu [26]. Demethylation of LINE-1 and Alu elements may induce insertion and/or homologous recombination and cause genomic alterations [27], and deregulate gene transcription [28]. These repeated elements are heavily methylated and represent much of the methylation in normal human genome [26], which has been shown to correlate with global genomic DNA methylation [26,29].

The aims of the study were to study how prenatal PFASs exposures interfere with global DNA methylation levels estimated by LINE-1 and Alu repeated elements and if the PFASs exposure related adverse birth outcomes were mediated by aberrant DNA methylation patterns.

## 2. Materials and Methods

### 2.1. Study Subjects and Questionnaire Data

Details of the study have been described previously [30]. Briefly, the study subjects were from the Taiwan Birth Panel Study (TBPS), a birth cohort study that was conducted at one medical center in Taipei and one local hospital and two clinics in New Taipei City from April 2004 to January 2005. An in-person interview was administered by trained interviewers by using structured questionnaires within three days of delivery. Structured prenatal and postnatal questionnaires were used to obtain information of parental socio-demographic characteristics, lifetime residential history, and potential environmental exposure. Medical records were used to extract in formation of infant sex, gestational age, and birth outcomes. A total of 486 sets including mother and their children participated and completed interview. 

### 2.2. Sample Collection

Informed consents were obtained from mothers before delivery to participate in the study. Maternal blood before delivery (10) mL and umbilical cord blood (10) mL at birth, and maternal urine (30) mL after delivery were collected. Maternal and cord blood samples were collected at birth in EDTA tubes and separated into two tubes of whole blood and four tubes of serum. Genomic DNA was isolated from cord blood using chemagic DNA blood kit special (Chemagen, Aachen, Germany) following the manufacture’s protocol. The samples remain frozen under −80 °C until laboratory analyses were done. The study has been approved by the Independent Ethics Committee of National Taiwan University Hospital (ethical code: 201307078RINB).

### 2.3. Methylation Analysis

Blood leucocyte DNA was extracted and bisulfite treated and PCR amplified. Bisulfite conversion of 500 ng of genomic DNA from blood was performed using EZ DNA Methylation reagents (Zymo Research, Orange, CA, USA). Alu and LINE-1 methylation analyses were conducted following the method developed by Yang et al. [26]. Four CpG sites were detected in LINE-1 and three CpG positions in Alu. A validated commercial kit for primers from Qiagen (Qiagen, Crawley, UK) were used for LINE-1 methylation measurements. The primer set of Alu (Eurofins MWG Operon, Ebersberg, Germany) was performed using previously published methods [26,31]. The primer sequence and sequence to analysis can be found at Appendix A Table A1. Built-in controls were used to verify bisulfite conversion. Pyrosequencing was performed by the PyroMark Q96 Pyrosequencing System.

### 2.4. Exposure Measurements

The concentration of cotinine and perfluorinated compounds were measured in plasma samples from cord blood. Detail of the analyses and the results can be found in the previous publication [32]. In brief, 12 PFASs were measured by ultra-high-performance liquid chromatography/tandem mass spectrometry method using the Waters ACQUITY UPLC system (Waters Corporation, Milford, MA, USA) coupled with a Waters Quattro Premier XE triple quadrupole mass spectrometer, including PFHxA, PFHpA, PFHxS, PFOA, PFOS, PFNA, PFDeA, PFUA, Me-PFOSA-AcOH, Et-PFOSA-AcOH, PFOSA, PFDoA in cord blood. All the laboratory experiments were analyzed by investigators blinded to the characteristics of study subjects. Four PFASs, PFOS, perfluoroundecanoic acid (PFUA), PFOA and perfluorononanoic acid (PFNA) with detection rates higher than 60% were included in the analyses of this study (the detection rates were 98.9%, 85.1%, 81.9%, and 67.6%, respectively). The limits of quantitation (LOQs) of PFOS, PFUA, PFOA and PFNA were 1.58, 0.22, 0.84, and 3.1 ng/mL, respectively. We allocated values equal to one-half of the quantitation limit for the PFASs levels less than their LOQs. Cotinine, one of the most sensitive biomarker of nicotine for smoking exposure, was analyzed in maternal and cord blood samples by High-Performance Liquid Chromatography (PerkinElmer, Boston, MA, USA) coupled to a Triple-Quadruple Tandem Mass Spectrometer (API 3000TM, Applied Biosystems, Foster, CA, USA).

### 2.5. Statistical Analysis

A total of 363 study participants with enough input of DNA amounts were included in our data analyses. The data were analyzed using the SAS^®^ 9.4 software (Cary, NC, USA) and R version 3.3.1 (The R Project for Statistical Computing, Vienna, Austria; http://www.r-project.org/). The degree of methylation was expressed as percentage of methylated cytosines (% 5mC). The PFASs exposures levels were natural log transformed due to right-skewed distribution. Methylation levels were evaluated by continuous and dichotomized status (i.e., *below or above medium)*. The methylation levels above medium were treated as reference groups. Previous study of the study population has reported PFOS exposure is associated with several adverse birth outcomes including preterm birth, low birth weight, and small for gestational age [10]. Preterm birth was defined as a gestational age <37 weeks. Low birth weight was defined as a birth weight <2500 g. A birth weight below the 10th percentile for gestational age was classified as small for gestational age according to the percentile scale derived from national Taiwanese data [33]. The associations of these adverse birth outcomes and methylation levels were evaluated. Among the detected significant PFASs exposures-DNA methylation levels and DNA methylation levels-adverse birth outcomes associations, mediation analyses [34] were conducted to determine the extent to which the association between PFASs exposures and adverse birth outcomes were mediated by DNA methylation [35].

Selected principal characteristics were evaluated by χ^2^, Fisher exact, and t tests, as appropriate. Univariable and multivariable regression models were used to study the exposures effects on DNA methylation levels and the associations between DNA methylation levels and adverse birth outcomes. Several variables were considered as potential confounders and were adjusted for in the multiple regression models: parental education level, maternal BMI, maternal age, delivery method (vaginal delivery or cesarean section), parity, infant sex, gestational age, and cotinine levels (natural log transformed) in cord blood. The variables change on the associations of interest more than 10% were considered as confounders and were adjusted in the final model. All reported *p*-values are from two-sided tests. A *p*-value < 0.05 was considered to be statistically significant.

## 3. Results

The characteristics of the study population are presented in Table 1. Among the 486 study participants, 363 of them with enough DNA samples available were included in the analyses. The mean methylation levels (SD) for LINE-1 and Alu were 80.16% (3.99) and 20.71% (1.72), respectively. The participants included in the analyses had younger maternal age at delivery, higher cord blood cotinine level, shorter gestational age and higher percentage of preterm birth compare to the ones excluded (Table 1). There were no statistically significant differences in maternal BMI, parental education level, delivery method, infant’s sex, and birth weight between the participants included and excluded from the current analyses. 

The levels of PFOS, PFOA, PFNA, and PFUA from cord blood are summarized in Table 2. The subjects included in the analyses had higher PFASs exposure measurements compared to the ones excluded. The distribution of the four PFASs measurements in male and female infant participants are summarized in Table 3. Female infant participants had higher PFUA measurements than male. There was no statistically significant difference of PFOS, PFOA, and PFNA between male and female participants. 

The associations between PFASs exposures and DNA methylation levels are summarized in Table 4. The crude univariable regression models represent the associations without adjusting for potential confounders. In the univariable models, we found that LINE-1 and Alu methylation levels decreased with the increase of PFOS exposure measurement (β = −0.76, 95% CI = (−1.48, −0.05), *p* = 0.03 and β = −0.27, 95% CI = (−0.54, −0.01), *p* = 0.04, respectively). For logistic regression, when dichotomized methylation level by medium and used the methylation level above medium as the reference group, each unit increase in the natural log-transformed PFOS and PFUA exposures were associated with ORs of 1.71 (95% CI = (1.19, 2.44); *p* = 0.003) and 1.26 (95% CI = (1.04, 1.52); *p* = 0.01) in association with low Alu methylation level. In multivariable models after adjusting for parental education level, maternal BMI, maternal age, delivery method (vaginal delivery or cesarean section), parity, infant sex, gestational age, and cotinine levels in cord blood, Alu methylation levels decreased with the increase of PFOS exposure measurement (β = −0.33, 95% CI = (−0.63, −0.02), *p* = 0.03). When dichotomized methylation level by medium and used the methylation level above medium as the reference group, each unit increase in the natural log-transformed PFOS exposure was associated with an adjusted OR (aOR) of 1.72 (95% CI = (1.03, 2.88); *p* = 0.02) in association with low Alu methylation level. The association between PFOS and low Alu methylation level was remained significant after adjusting for potential confounder effects while PFUA was not. We did not observe significant effects between PFOA, PFNA, PFUA and methylation levels in the multivariable regression models. 

Previous study of the study population has reported PFOS exposure is associated with several adverse birth outcomes including preterm birth, low birth weight, and small for gestational age [10]. We also computed odds ratios for the association between the adverse birth outcomes and low Alu, the DNA methylation levels we found significantly associated with PFASs exposures after controlling for potential confounders. Higher proportion of the preterm-born neonates had low Alu methylation (69%) compared with term-born ones (48%) (*p* = 0.01 by χ^2^ test). Low Alu methylation was associated with an OR 2.46 (95% CI = (1.20, 5.03)). The significant association did not remain after adjusting for potential confounders. No significant association between small for gestational age, low birth weight, and DNA methylation level was observed. We further estimated the proportion of PFOS-related adverse birth outcome that is mediated by variation in DNA methylation levels. In the mediation analyses, we did not find significant mediation effect by low Alu methylation level among PFOS exposure and preterm birth.

## 4. Discussions

In the present study, we examined the prenatal PFASs exposures effects at global DNA methylation estimated in Alu and LINE-1 repeated elements in a birth cohort study in Taiwan. Our findings suggest that prenatal PFOS exposure was associated with low Alu repeated elements after controlling for potential confounders. 

Study in rats has shown that global methylation level was significantly decreased among the high PFOS in utero exposure group (2.0 mg/kg/day) but not among the medium (0.6 mg/kg/day) and low exposure groups (0.1 mg/kg/day)) [36]. To our knowledge, there were only few epidemiology studies have reported prenatal PFOA and PFOS exposure effects in association with global DNA methylation level changes [37,38]. Among the 30 babies from Baltimore THREE (Tracking Health Related to Environmental Exposures) Study, Guerrero-Preston et al. [37] reported a negative association between global DNA methylation level by using ELISA-based method and PFOA and PFOS measurements in cord blood. The association of PFOA was borderline statistical significance (*p* = 0.06) while PFOS was not. Among the 177 Mother–child pairs from the Hokkaido Study, Kobayashi et al. [38] reported no statistically significant associations were observed between LINE1 methylation levels with either PFOA or PFOS (adjusted β = −0.15 and 0.05, *p* = 0.244 and 0.761, respectively for PFOA and PFOS exposure effect). The PFOS and PFOA levels by means of our study were slightly higher than Guerrero-Preston et al. (5.8 and 1.8 ng/,mL respectively) [37] and Kobayashi et al. (5.7 and 1.6 ng/mL respectively) [38]. Alu methylation levels were not measured in either of the two studies. Among the studies on adult populations, sperm Alu methylation levels and PFASs exposures has been reported by Leter et al. [39] among 262 fertile men from three independent populations in Greenland, Poland and Ukraine. PFOS exposure was associated with decreased Alu methylation levels, though the associations were not statistically significant.

There are several strengths of this present study including the prospective cohort design to minimize recall bias. The current study also collected comprehensive information to help us adjust for several potential confounders/effect modifiers reported by previous studies. Besides, instead of using maternal self-reported data, cotinine measurements were used to control for smoking related confounding effect and medical records were used to collect perinatal information such as birth weight and gestational age to improve data reliability. There are only few birth cohort with PFASs exposure information available, our findings add to the understanding of the PFASs mechanisms. There are several potential limitations of the study. First, the participants included in the current analyses had higher PFASs and cotinine measurements than the excluded participants. Our results may not be able to be generalized to the population with lower exposure levels. Second, the methylation alterations may be tissue-specific, though blood DNA is widely used as a surrogate and precursor to test for the exposure effects or disease markers. The interpretation of our findings may be limited as an early biomarker for biological processes that shows systemic influence. Further, if PFASs exposure is associated with types of leukocytes present in peripheral blood, leukocyte type may confound the associations between PFASs exposures and global methylation levels. Although Alu methylation levels was not found to be significantly associated with the differential white blood cell count in core blood [40] and in adults’ blood [41]. The Alu elements occupy 11% of the human genome [42] and are not randomly distributed in the genome. They are frequently enriched in gene-rich regions [43]. The Alu elements may affect gene expression through polyadenylation [44], splicing [5,6,7] and adenosine deaminase that acts on RNA editing [8,9,10]. 

## 5. Conclusions

PFASs have been suggested to be carcinogenic without DNA damage [13], suggesting a potential epigenetic regulation. Our findings support that prenatal PFOS exposure may be associated with low Alu methylation level.

## Figures and Tables

**Table 1:** Distribution of study subjects by demographic and risk factors.

Characteristics ^a^	Total (*N* = 486)	Included (*N* = 363)	Excluded (*N* = 123)
Maternal age at delivery (years) *	30.8 (4.7)	30.4 (4.7)	32.1 (4.3)
Maternal BMI (kg/m^2^)	20.9 (3.1)	21 (3.3)	20.6 (2.4)
Parental education level ^b^			
Not senior high school graduated (%)	3 (0.6)	3 (0.8)	0 (0)
Senior high school graduated (%)	24 (4.3)	19 (5.3)	2 (1.6)
Four–year college /university and above (%)	485 (95.1)	340 (93.9)	121 (98.4)
Delivery method, vaginal (%)	60.1	60.5	58.9
Infants			
Boys (%)	50.6	49.5	54
Birth weight (g)	3157.9 (476.6)	3141.7 (490.0)	3204.7 (433.9)
Gestational age (weeks) *	38.5 (1.7)	38.4 (1.8)	38.8 (1.5)
Low birth weight (<2500 g) (%)	28 (5.8)	25 (6.9)	3 (2.4)
Small gestational age (%)	32 (6.6)	23 (6.4)	9 (7.3)
Preterm birth (<37 weeks) (%) *	42 (8.6)	39 (10.7)	3 (2.4)
Cord blood			
Cotinine (ng/mL) *	3.4 (19.2)	4.5 (22.3)	0.8 (5.8)
LINE-1 methylation (%)		80.2 (3.99)	
Alu methylation (%)		20.7 (1.72)	

^a^ All characteristics are expressed as mean (SD) or percentage. ^b^ Highest education level of either parent. * *p* < 0.05 by comparing included and excluded participants.

**Table 2:** Distribution of PFASs exposure measurements in cord blood.

Exposure ^a^ (ng/mL)	Detection Limit (ng/mL)		Mean	(SD)	GM	(GSD)	Min.	Median	Max.
PFOS *	0.066	Total	7.66	(7.34)	5.97	(1.95)	0.11	5.67	67.92
		Included	7.80	(7.66)	6.07	(1.93)	1.08	5.70	67.92
		Excluded	7.24	(6.34)	5.69	(2.04)	0.11	5.61	48.36
PFOA *	1.23	Total	2.57	(2.38)	1.84	(2.24)	0.75	1.86	17.40
		Included	2.88	(2.55)	2.05	(2.28)	0.75	2.12	17.40
		Excluded	1.71	(1.52)	1.33	(1.93)	0.75	1.02	9.45
PFNA *	0.67	Total	6.29	(8.39)	2.38	(4.70)	0.38	3.00	63.87
		Included	7.11	(8.98)	2.77	(4.74)	0.38	3.86	63.87
		Excluded	3.93	(5.81)	1.54	(4.23)	0.38	1.36	39.87
PFUA *	2.4	Total	16.9	(15.88)	10.12	(3.11)	1.50	13.50	102.36
		Included	18.10	(17.14)	10.63	(3.20)	1.50	14.18	102.36
		Excluded	13.45	(10.84)	8.77	(2.84)	1.50	10.41	45.75

Abbreviations: GM, geometric mean; GSD, geometric standard deviation; PFASs, perfluoroalkyl substances; PFNA, perfluorononanoic acid; PFOA, perfluorooctanoic acid; PFOS, perfluorooctyl sulfonate; PFUA, perfluoroundecanoic acid. ^a^ Concentration values below the limits of quantitation were set to be 1/2 limit of quantitation. * *p* < 0.05 by comparing included and excluded participants.

**Table 3:** Distribution of exposure measurements in cord blood by infant’s sex.

Exposure ^a^ (ng/) mL	Infant’s Sex	Mean (SD)	GM (GSD)	Min.	Median	Max.
PFOS	Boy	8.44 (9.52)	6.08 (2.09)	1.83	5.60	67.92
	Girl	7.05 (4.99)	5.85 (1.81)	1.08	5.70	38.85
PFOA	Boy	2.90 (2.51)	1.84 (2.22)	0.75	2.24	13.86
	Girl	2.87 (2.60)	1.83 (2.27)	0.75	2.10	17.40
PFNA	Boy	6.87 (7.94)	2.27 (4.83)	0.38	3.95	34.56
	Girl	7.45 (9.95)	2.51 (4.57)	0.38	3.90	63.87
PFUA *	Boy	16.42 (17.33)	8.57 (3.25)	1.50	11.07	98.76
	Girl	19.82 (16.90)	12.08 (2.89)	1.50	16.62	102.36

Abbreviations: GM, geometric mean; GSD, geometric standard deviation; PFASs, perfluoroalkyl substances; PFNA, perfluorononanoic acid; PFOA, perfluorooctanoic acid; PFOS, perfluorooctyl sulfonate; PFUA, perfluoroundecanoic acid. ^a^ Concentration values below the limits of quantitation were set to be 1/2 limit of quantitation. * *p* < 0.05.

**Table 4:** The associations between PFASs exposures and DNA methylation levels.

DNA Methylation (% 5mC)	Exposure ^a^	Crude Linear Regression Model			Multiple Linear Regression Model ^b^			Crude Logistic Regression Model ^c^			Multiple Logistic Regression Model ^bc^		
		β	(95% CI)	*p*-Value	β	(95% CI)	*p*-Value	OR	(95% CI)	*p*-Value	OR	(95% CI)	*p*-Value
LINE-1	PFOS	**−0.76**	**(−1.48, −0.05)**	**0.03**	−0.67	(−1.61, 0.26)	0.16	1.19	(0.85–1.66)	0.31	1.08	(0.69–1.70)	0.48
	PFOA	0.55	(−0.02, 1.12)	0.06	0.41	(−0.38, 1.20)	0.31	0.84	(0.65–1.10)	0.21	0.97	(0.67–1.40)	0.97
	PFNA	0.03	(−0.27, 0.33)	0.84	−0.07	(−0.48, 0.35)	0.75	1.02	(0.89–1.17)	0.78	1.13	(0.93–1.36)	0.17
	PFUA	−0.36	(−0.77, 0.04)	0.08	−0.47	(−1.02, 0.09)	0.10	1.10	(0.91–1.33)	0.31	1.06	(0.82–1.37)	0.56
Alu	PFOS	**−0.27**	**(−0.54, −0.01)**	**0.04**	**−0.33**	**(−0.63, −0.02)**	**0.03**	**1.71**	**(1.19–2.44)**	**0.003**	**1.72**	**(1.03–2.88)**	**0.02**
	PFOA	0.04	(−0.18, 0.25)	0.73	−0.03	(−0.28, 0.23)	0.82	0.89	(0.68–1.17)	0.40	1.10	(0.75–1.62)	0.48
	PFNA	−0.02	(−0.14, 0.09)	0.68	0.03	(−0.11, 0.16)	0.68	0.96	(0.83–1.11)	0.57	1.01	(0.83−1.24)	0.88
	PFUA	−0.05	(−0.2, 0.09)	0.47	−0.03	(−0.21, 0.14)	0.90	**1.26**	**(1.04–1.52)**	**0.01**	1.13	(0.86–1.47)	0.26

^a^ Exposure levels are natural log-transformed; ^b^ Multivariable regression model adjusted for parental education level, maternal BMI, maternal age, delivery method (vaginal delivery or cesarean section), parity, infant sex, gestational age, and cotinine levels in cord blood; ^c^ The associations were estimated by dichotomized methylation status (i.e., below or above medium). The methylation levels above medium were treated as reference groups.

## Appendix A

**Table A1:** Primers.

ID	Forward Primer (5′ to 3′)	Reverse Primer (5′ to 3′)	Sequencing Primer (5′ to 3′)	Sequence to Analysis
Global methylation analysis				
PyroMark Q96 CpG LINE-1	Commercial kit	Commercial kit	Commercial kit	TTC/TGTGGTGC/TGTC/TGTTTTTTAAGTC/TGGTTT
Alu	bio-TTTTTATTAAAAATATAAAAATT	CCCAAACTAAAATACAATAA	AATAACTAAAATTACAAAC	A/GC/TA/GC/TAGCCACCA
